# Giant Dipole Moments: Remarkable Effects Mono‐, Di‐, and Tri‐ Hydrated 5,6‐Diaminobenzene‐1,2,3,4‐Tetracarbonnitrile

**DOI:** 10.1002/jcc.70105

**Published:** 2025-04-18

**Authors:** Katherine Stanley, R. Houston Givhan, Justin M. Turney, Henry F. Schaefer

**Affiliations:** ^1^ Center for Computational Quantum Chemistry University of Georgia Athens Athens Georgia USA

**Keywords:** dipole, energies, hydrogen bonding, solvent

## Abstract

The molecule 5,6‐diaminobenzene‐1,2,3,4‐tetracarbonnitrile (MOI) was first synthesized by Müllen and coworkers in 2016 and boasts an ultrastrong dipole moment of 14.1±0.7 Debye in THF. Gas phase DFT computations do not fully reflect this ultrastrong dipole moment, demonstrating the role of solvent in increasing this dipole moment. Here, we investigate the effect of solvent molecule position on the dipole moment of this species, computationally examining systems with giant dipole moments. These systems are optimized in the gas phase with the B3LYP functional, employing the aug‐cc‐pVTZ and def2‐TZVP basis sets, as well as the B3LYP‐D3BJ/aug‐cc‐pVTZ functional in Orca. Single point DLPNO‐CCSD/aug‐cc‐pVDZ results were obtained from Orca and Psi4, as well as DLPNO‐CCSD(T)/CBS information from Psi4. Additionally, these are compared to the dipole moments of di‐ and tri‐hydrated systems, and the SMD models for THF and water at the B3LYP/aug‐cc‐pVTZ level of theory. The dissociation energies, HOMO‐LUMO energy gaps, and dipole moments are presented. These metrics show the nh1nh1′ THF system boasts the largest dissociation energy and dipole moment of the singly solvated systems, due to its strong hydrogen bonding. The importance of solvent placement is highlighted and may guide the synthesis of macromolecules or organic frameworks incorporating the MOI or MOI‐like subunits. Remarkably, a single solvent molecule provides a good model for the difference between the gas phase and solvated species. The predicted gas phase dipole moments computed with B3LYP/aug‐cc‐pVTZ for the MOI, its monohydrated complex, dihydrated complex, and its trihydrated complex are 9.6, 14.2, 16.0, and 16.8 Debye, respectively.

## Graphical abstract

Solvent placement has a significant impact on the dipole moment of neutral species, which is especially important in the case of ultrastrong dipole moments. Using water and THF, the dissociation energies, relative energies, HOMO‐LUMO gaps, dihedral angles, and dipole moments of 5,6‐diaminobenzene‐1,2,3,4‐tetracarbonnitrile are accessed.

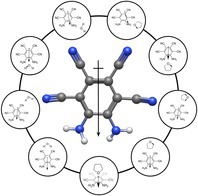

## 1 Introduction

Analyzing the dipole moments of neutral species can play a critical role in understanding the nature of the species. This importance is highlighted in the case of ultrastrong dipole moments, defined by a high level of charge separation between the positive and negative charges of the system [1]. When synthesizing macromolecules, the dipole moment and polarizability of the utilized subunits will affect the final product [2]. Furthermore, ultrastrong dipole moments hold potential applications in organic ferroelectrics, as well as nonlinear optics [3, 4]. Higher dipole moments are additionally associated with an increase in dielectric constants, which has a known effect on reaction rates and the stability of activated complexes [5, 6].

The simplest approximation for dipole moment can be expressed by the equation:

μ=q×r

where μ is the dipole moment, q the charge, and r the distance [7]. The three primary methods of modeling solute compounds include gas phase computations, implicit solvation, and explicit solvation.

Gas phase ab initio and DFT computations may be considered the simplest of these three methods, as they do not account for solute‐solvent interactions. It is well known that a polar solvent can induce a dipole moment in a nonpolar molecule [7], and it has been shown that polar molecules in a polar solvent may also see increases in dipole moments [8]. These gas phase computations may thus underestimate the dipole moment of solutes in polar solvents.

Implicit solvation, another common technique for modeling solutes, is the method of using a continuum model to approximate the bulk effects of the solvent. This is often done by decoupling the polar and apolar interactions of the solvent. The polar contribution is approximated by the Poisson–Boltzmann equation, which is dependent on the dielectric constant of the solvent [9]. This approximation greatly decreases the computational cost of these calculations relative to explicit solvation. Furthermore, this approach may be better equipped to account for the long‐range effects the solvent will have on the solute [10, 11]. Despite these benefits, there is an intrinsic limitation in most implicit models when examining charged or radical species [10] or systems involved with strong hydrogen bonding [12]. Thus, additional techniques are required to describe these more complex systems.

Finally, explicit solvation involves a gas phase computation of a solute, often with the addition of up to four solvent molecules surrounding the solute [13]. This practice is commonly used in modeling ionic species due to their increased interactions with the solute [7]. The solvation shell, an area around the solute where the solute molecule's presence disrupts the solvent's bulk structure, will generally increase as the polarity of the solute increases [7]. Thus, when modeling ionic species, an implicit solvation model may fail to adequately describe the solvation shell, which alters many of the characteristics of the solute, including dipole. Additionally, information regarding the solute–solvent interactions can be gained through explicit solvation, as well as a better understanding of how solvent location affects the nature of the solute. A primary drawback to explicit solvation is its high computational cost. Additionally, there is the question of chemical stability, specifically when using a low number of solute molecules.

In 2016, Müllen and coworkers synthesized the system 5,6‐diaminobenzene‐1,2,3,4‐tetracarbonnitrile, our molecule of interest (MOI) in this research. This MOI is a neutral species and is classified as having an ultrastrong dipole moment. It was synthesized through oxidative bromination, followed by palladium‐assisted cyanation [14].

The experimental dipole measurement for the MOI was carried out using a Novocontrol Alpha frequency analyzer with dilute solutions of the MOI in THF. Dielectric permittivity was related to the concentration of the MOI, and the experimental dipole was determined from the slope of this relation. This yielded an experimental dipole moment of 14.1±0.7 Debye. In contrast, our gas phase B3LYP/aug‐cc‐pVTZ computation of the MOI presented in this research showed a dipole moment of 9.6 Debye. This discrepancy might be attributed to the effects of the solvent. When analogous dipole calculations were performed using the SMD implicit solvation model, there was a 42% increase in dipole from the gas phase computations [14]. Thus, it is evident that the solvent had significant effects on the MOI.

Given this discrepancy, our objective is to investigate the nature of the MOI, given the addition of a single solvent molecule to maximize this ultrastrong dipole. Furthermore, it is questioned whether the addition of a single solvent molecule could reasonably approximate many of the effects of the solvent on the MOI in a computationally efficient manner. Moreover, just as the dipole moment is affected by the introduction of the solvent, many other characteristics of the MOI may also be altered by the solvent, such as dihedral angles, HOMO‐LUMO energy gaps, and the stability of the solute–solvent system.

Using THF and water, the effects of solvent on the MOI were accessed here at various geometrical positions, providing greater insight into the interactions between the solute and solvent. These systems were analyzed at various levels of theory and they highlight the importance of solvent position in increasing dipole moments.

## 2 Methods

Due to the symmetry of the MOI, four potential locations for the solvent relative to the MOI were initially identified. Each system was optimized using the B3LYP [15, 16, 17, 18] functional with the aug‐cc‐pVTZ [19, 20] and def2‐TZVP [21] basis sets, as well as the B3LYP‐D3BJ/aug‐cc‐pVTZ [22] functional using Orca 6.0 [23].

Structural information was obtained from the B3LYP/aug‐cc‐pVTZ optimized systems, as it has been shown that this combination is well equipped to describe dipole moments [24]. Natural Bond Orbital (NBO) [25] analysis was completed on each system. For natural atomic charge analysis and bond order information, see the Tables S13 and S14. The Conformer‐Rotamer Ensemble Sampling Tool (CREST) of Grimme [26] was applied to each system, to ensure that all chemically viable structures were accounted for.

Finite difference perturbed Hamiltonian dipole moments obtained with DLPNO‐CCSD(T)/CBS [27, 28, 29, 30, 31] at the TightPNO convergence level were computed, with the following equation used to perform the CBS extrapolation. This is done using the implementation of DLPNO‐CCSD(T) in Psi4 [32, 33].

$$\begin{array}{c} \begin{array}{l} E_{\text{DLPNO}-\text{CCSD}\left(T\right)/\mathrm{CBS}}=\left(E_{\text{DLPNO}-\text{CCSD}\left(T\right)/\mathrm{aug}-\mathrm{cc}-\text{pVDZ}}-E_{\text{DLPNO}-\mathrm{MP}2/\mathrm{aug}-\mathrm{cc}-\text{pVDZ}}\right)\\ \;+E_{\mathrm{MP}2/\mathrm{aug}-\mathrm{cc}-\mathrm{pV}\left[\mathrm{TQ}\right]Z} \end{array} \end{array}$$

With Helgaker's extrapolation used for MP2

(1) $$E_{\mathrm{MP}2/\mathrm{CBS}}\approx \frac{E_{\mathrm{MP}2/\mathrm{aug}-\mathrm{cc}-\text{pVQZ}}-E_{\mathrm{MP}2/\mathrm{aug}-\mathrm{cc}-\text{pVTZ}}}{4^{3}-3^{3}}$$

Analytic dipole moments [34] at the DLPNO‐CCSD/aug‐cc‐pVDZ [29, 31] level of theory were computed with Orca [35], using an unrelaxed density matrix. Additionally, single point DLPNO‐CCSD(T)/CBS energies were obtained on Psi4, as well as single point DLPNO‐CCSD/aug‐cc‐pVTZ energies on Orca.

Dissociation energies for each system were computed in the following manner:

$$E_{\mathrm{Dis}}=E_{\mathrm{MOI}}+E_{\text{solvent}}-E_{\text{System}}$$

where $E_{\mathrm{MOI}}$ and $E_{\text{solvent}}$ are the energies of the MOI and the solvent molecule infinitely separated. $E_{\text{System}}$ is the energy of the entire solute‐solvent system.

SMD optimizations of the MOI were performed in Orca with B3LYP/aug‐cc‐pVDZ using the water and THF solvent models. Additionally, di‐ and tri‐hydrated systems with the MOI were computed at B3LYP/aug‐cc‐pVTZ, with a particular emphasis on maximizing the dipole moment.

## 3 Results

### 3.1 Geometries

**FIGURE 1:**
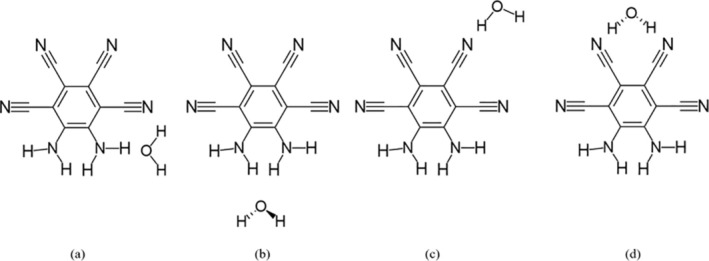
Systems of the MOI with one attached water molecule were examined: (a) nh1, (b) nh1nh1′, (c) cn2, (d) cn2cn2′.

**FIGURE 2:**
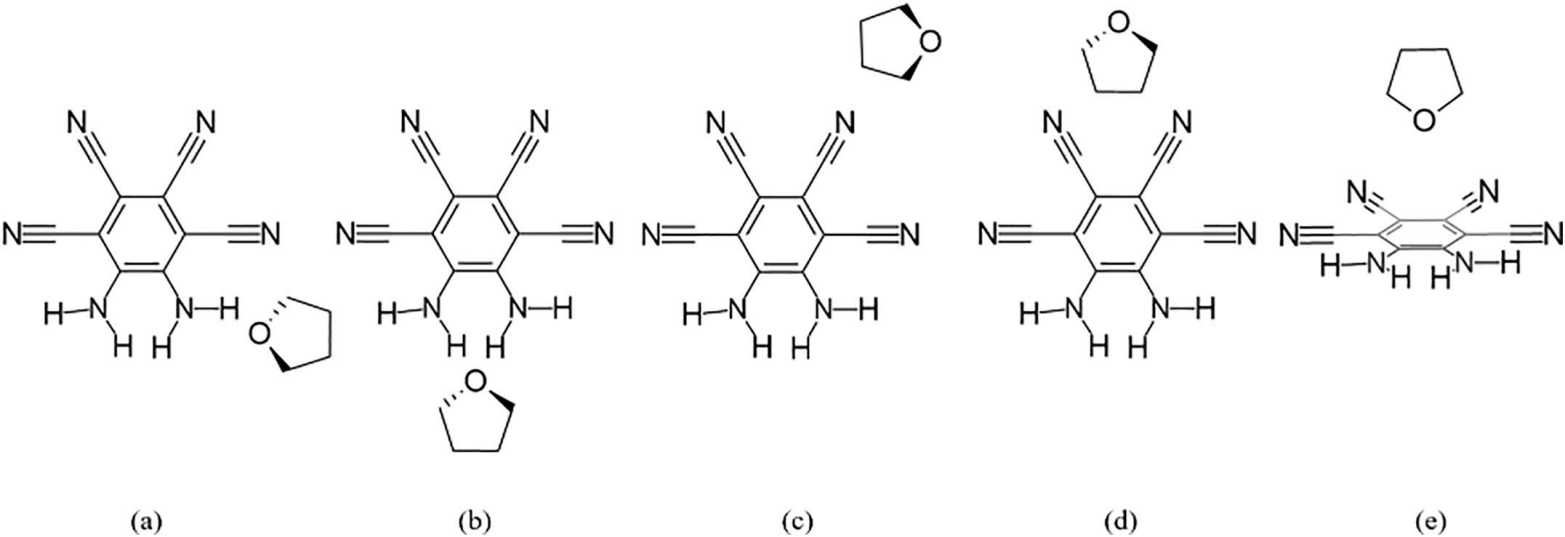
Systems of the MOI with one attached THF molecule were also examined: (a) nh1, (b) nh1nh1′, (c) cn2, (d) cn2cn2′, (e) ring.

Initial geometries were constructed to take advantage of the symmetry of the MOI. Four systems were found with water, presented in Figure 1, and five with THF, presented in Figure 2. The fifth structure has the THF placed over the MOI ring. CREST analyses found that across the water systems, the nh1nh1′ position was preferred, with the nh1 system being the second‐most favorable. For THF, the energetically favorable systems were found at the nh1nh1′, nh1, and ring positions. In the gas phase, the MOI presented a C_2_ symmetry. In all water systems, the water molecule held its $C_{2v}$ symmetry. Across the THF systems, the THF molecule showed a C_2_ symmetry, with the sole exception of the ring position, in which the THF molecule showed $C_{s}$ symmetry.

#### 3.1.1 Dihedral Angles

**FIGURE 3:**
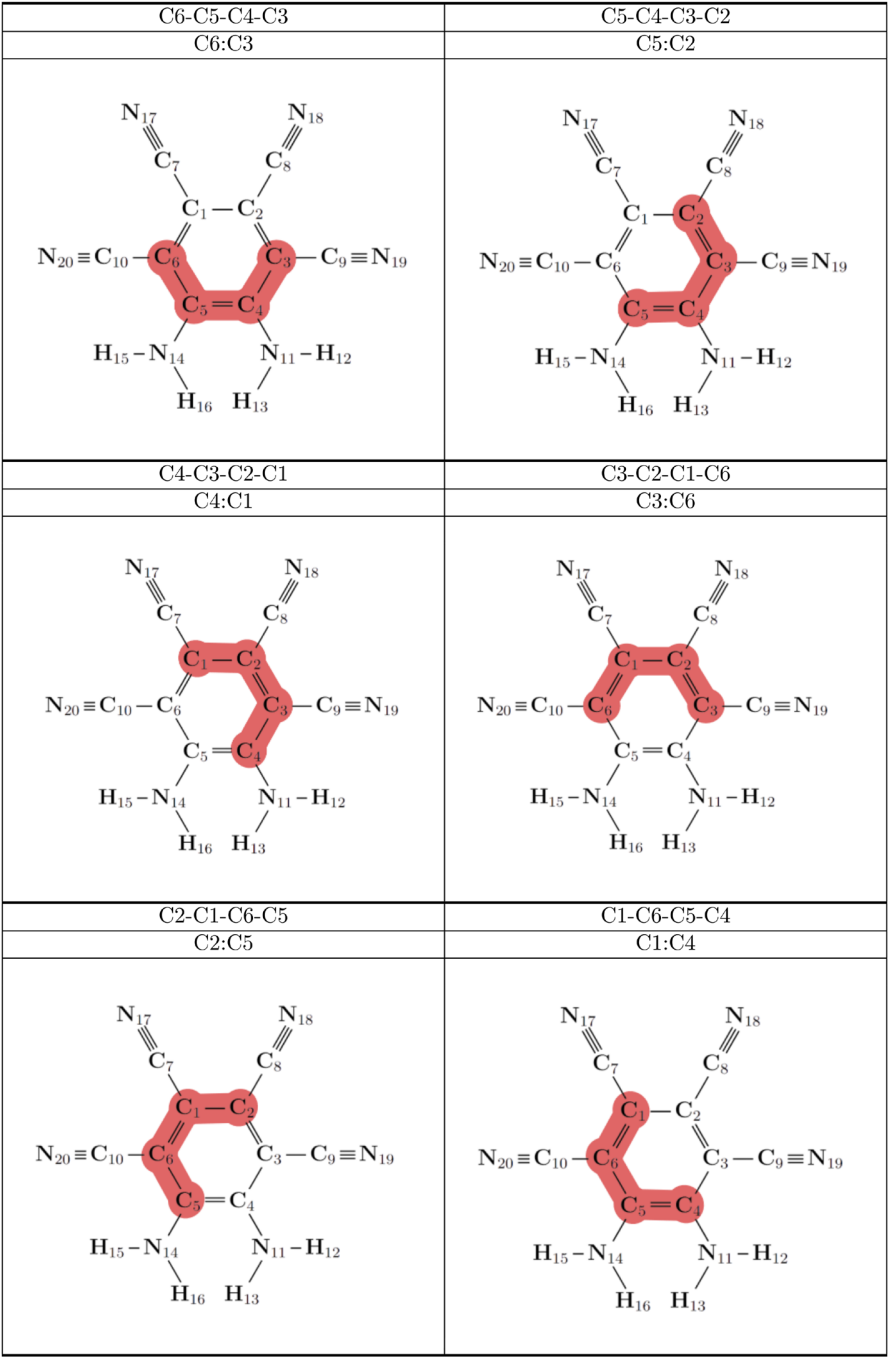
Dihedral angles of the MOI presented in Table 1 are highlighted here in red. Each dihedral describes the four adjacent carbon atoms on the ring of the MOI.

From Table 1, it can be seen that the dihedral angles in the ring of the MOI (Figure 3) remain relatively flat regardless of solvent location, which is perhaps expected of an aromatic system. It may be noted that the nh1nh1′ position in both the THF and water systems holds the lowest dihedral values; however, when each of these systems is optimized with the dihedral angles in Table 1 constrained to zero, no system presents a difference in energy greater than 1 kcal mol^−1^, indicating the ring is essentially planar across all systems.

**TABLE 1:** Dihedral angles in degrees of the MOI with the addition of the solvent, attached at the various positions. Each dihedral value relates four adjacent carbon atoms on the MOI ring, depicted in Figure 3.

System		C6:C3	C5:C2	C4:C1	C3:C6	C2:C5	C1:C4
MOI		3.2	−2.0	−0.6	1.9	−0.6	−1.9
nh1	Water	3.4	−2.2	−0.5	2.0	−0.8	−2.0
	THF	3.9	−2.9	−0.1	2.1	−1.0	−2.0
nh1nh1′	Water	0.4	−0.2	−0.2	0.4	−0.2	−0.2
	THF	0.2	−0.1	0.0	0.1	−0.1	−0.1
cn2	Water	3.2	−2.0	−0.6	1.8	−0.6	−2.0
	THF	3.2	−2.0	−0.5	1.8	−0.6	−2.0
cn2cn2′	Water	3.3	−1.9	−0.7	1.9	−0.5	−2.1
	THF	3.2	−2.0	−0.6	1.9	−0.6	−2.0
ring	Water						
	THF	−3.5	2.5	0.3	−2.1	1.0	1.8
SMD	Water	3.0	−2.1	−0.3	1.6	−0.6	–1.7
	THF	3.4	−2.2	−0.4	1.9	−0.7	–2.0

*Note:* Obtained from B3LYP/aug‐cc‐pVTZ optimized structures.

**FIGURE 4:**
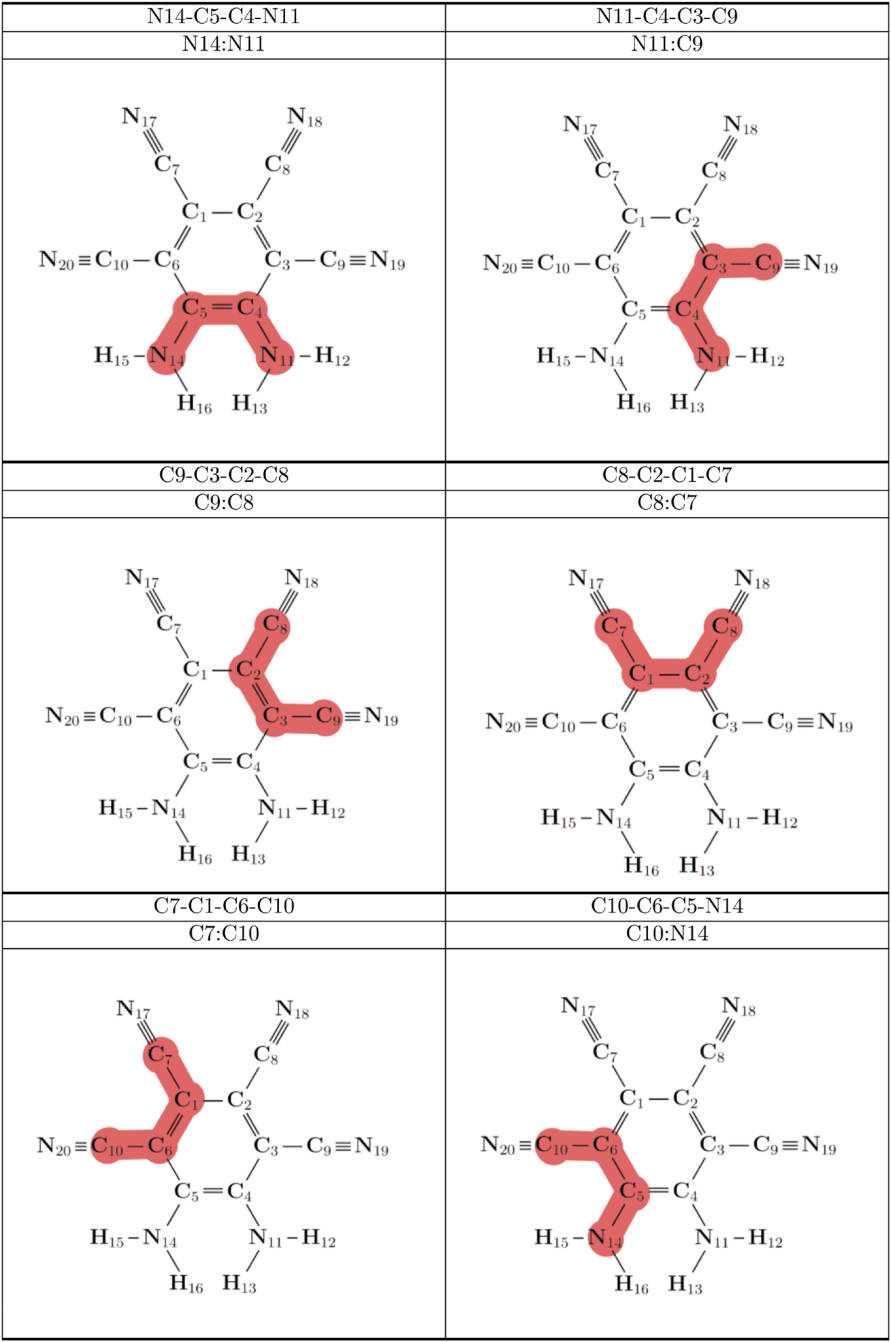
Dihedral angles of the MOI presented in Table 2 highlighted in red. Each dihedral contains two adjacent substituents of the MOI with the respective carbon atoms on the ring.

For each complex, it can be seen that the dihedral angles relating adjacent cyano substituents in Table 2, pictured in Figure 4, were the flattest, which would allow greater delocalization of π electrons. In contrast, the dihedral angles associated with amine substituents were typically higher, with the highest N14:N11 dihedral arising in the nh1nh1′ water system at −2.6°. It is also of note that for the nh1 and nh1nh1′ systems, the water solvent consistently predicts higher dihedral values for the N14:N11 and N11:C9 dihedral angles than is the case for THF. For the nh1nh1′ systems, the water solvent also showed a higher dihedral at the C10:N14 position than for THF.

**TABLE 2:** Dihedral angles in degrees for the MOI with the addition of the solvent, placed at the various positions.

System		N14:N11	N11:C9	C9:C8	C8:C7	C7:C10	C10:N14
MOI		−1.6	1.9	−1.2	0.2	−1.2	1.9
nh1	Water	−0.9	1.5	−1.4	0.3	−1.4	1.9
	THF	−0.6	0.4	−0.6	0.4	−1.4	1.9
nh1nh1′	Water	−2.6	1.6	−0.4	0.0	−0.4	1.7
	THF	−1.1	0.7	−0.2	0.0	−0.1	0.7
cn2	Water	−1.4	1.9	−1.2	0.2	−1.2	1.8
	THF	−1.5	1.9	−1.1	0.1	−1.2	1.9
cn2cn2′	Water	−1.4	2.0	−1.7	0.3	−0.8	1.8
	THF	−1.5	1.9	−1.2	0.2	−1.2	1.9
ring	Water						
	THF	1.2	−2.1	1.2	−0.4	1.8	−1.6
SMD	Water	−0.8	1.0	−1.0	0.6	−1.3	1.4
	THF	−0.9	1.3	−1.2	0.6	−1.4	1.6

*Note:* Each dihedral value relates adjacent substituents and the respective carbon atoms on the ring and can be seen in Figure 4. Obtained from B3LYP/aug‐cc‐pVTZ optimized structures.

### 3.2 Energies

#### 3.2.1 Dissociation Energies

It may be seen in Table 3 that the systems with the largest dissociation energies are the nh1 and nh1nh1′ positions, though which of these systems has the higher dissociation energy differs between the aug‐cc‐pVTZ and def2‐TZVP basis sets. At the B3LYP/aug‐cc‐pVTZ level of theory, the nh1nh1′ system holds a lower dissociation energy to the third decimal place. It is of note that all DLPNO calculations were single‐point energy calculations run on the B3LYP/aug‐cc‐pVTZ optimized structures. The cn2 and cn2cn2′ systems consistently present the smallest dissociation energies.

**TABLE 3:** Dissociation energies of the one water complexes in various positions in kcal mol^−1^. MOI refers to the MOI infinitely separated from the water and is set to zero.

Method	MOI	nh1	nh1nh1′	cn2	cn2cn2′
B3LYP/aug‐cc‐pVTZ	0.00	8.34	8.34	3.74	3.25
B3LYP/def2‐TZVP	0.00	9.64	9.82	3.88	4.17
B3LYP‐D3BJ/aug‐cc‐pVTZ	0.00	10.34	9.92	4.41	5.85
DLPNO‐CCSD(T)/CBS	0.00	9.85	9.16	4.31	5.04
DLPNO‐CCSD/aug‐cc‐pVDZ (Orca)	0.00	9.70	8.92	4.47	4.94
DLPNO‐CCSD/aug‐cc‐pVDZ (Psi4)	0.00	9.56	8.95	4.50	4.98

In Table 4, it can be seen that the nh1nh1′ THF system yields the largest dissociation energy across all levels of theory. The nh1 system shows the second‐largest dissociation energy of the THF systems. There is a notable increase of over 4 kcal mol^−1^ in dissociation energy for both the nh1 and ring complexes with the addition of the D3BJ dispersion correction, resulting in the nh1 THF system holding the second‐largest dissociation energy of both water and THF at this level of theory.

**TABLE 4:** Dissociation energies for the one THF complex at various positions in kcal mol^−1^.

Method	MOI	nh1	nh1nh1′	cn2	cn2cn2′	Ring
B3LYP/aug‐cc‐pVTZ	0.00	7.68	10.85	0.48	0.77	2.56
B3LYP/def2‐TZVP	0.00	8.04	11.10	0.49	0.80	2.69
B3LYP‐D3BJ/aug‐cc‐pVTZ	0.00	12.14	14.37	1.29	2.50	8.75
DLPNO‐CCSD(T)/CBS	0.00	11.27	12.90	1.40	2.18	6.75
DLPNO‐CCSD/aug‐cc‐pVDZ (Orca)	0.00	12.36	13.29	1.96	2.78	8.95
DLPNO‐CCSD/aug‐cc‐pVDZ (Psi4)	0.00	12.43	13.31	1.95	2.79	8.74

*Note:* MOI refers to the MOI infinitely separated from the THF and is set to zero.

#### 3.2.2 Relative Energies

As seen in Table 5, among the one water systems, the nh1 system displayed the lowest relative energy, though the nh1nh1′ system is very close, with less than a 0.01 kcal mol^−1^ difference at the B3LYP/aug‐cc‐pVTZ level of theory. It can be seen that the difference in relative energies between the nh1 and nh1nh1′ systems is accentuated with the single‐point calculations at higher levels of theory, but remains within 1 kcal mol^−1^.

**TABLE 5:** Relative energies of the one water systems in kcal mol^−1^.

Method	nh1	nh1nh1′	cn2	cn2cn2′
B3LYP/aug‐cc‐pVTZ	0.00	0.00^a^	4.60	5.09
B3LYP/def2‐TZVP	0.18	0.00	5.95	5.65
B3LYP‐D3BJ/aug‐cc‐pVTZ	0.00	0.41	5.93	4.49
DLPNO‐CCSD(T)/CBS	0.00	0.69	5.54	4.81
DLPNO‐CCSD/aug‐cc‐pVDZ (Orca)	0.00	0.78	5.23	4.76
DLPNO‐CCSD/aug‐cc‐pVDZ (Psi4)	0.00	0.61	5.06	4.58

*Note:* For each system, the energy of the lowest energy system under that level of theory is subtracted from the system.

^a^ The nh1nh1′ water system is nonzero to the thousandths place.

As seen in Table 6, across the THF systems, the nh1nh1′ position consistently shows the lowest relative energy, with the nh1 and ring system being the second and third lowest, respectively. There is a notable drop in relative energy with the addition of the D3BJ dispersion correction at the ring position, and it can be seen that single‐point energy calculations that account for correlation also predict a notable drop in relative energy for both the ring and nh1 positions.

**TABLE 6:** Relative energies of the one THF systems in kcal mol^−1^.

Method	nh1	nh1nh1′	cn2	cn2cn2′	Ring
B3LYP/aug‐cc‐pVTZ	3.17	0.00	10.37	10.08	8.29
B3LYP/def2‐TZVP	3.06	0.00	10.62	10.30	8.41
B3LYP‐D3BJ/aug‐cc‐pVTZ	2.23	0.00	13.09	11.87	5.63
DLPNO‐CCSD(T)/CBS	1.63	0.00	11.51	10.72	6.16
DLPNO‐CCSD/aug‐cc‐pVDZ (Orca)	0.92	0.00	11.33	10.51	4.34
DLPNO‐CCSD/aug‐cc‐pVDZ (Psi4)	0.88	0.00	11.36	10.52	4.57

*Note:* For each system, the energy of the lowest energy system under that level of theory is subtracted from the system.

#### 3.2.3 Homo‐Lumo Gaps

While the focus of this paper is on the high Dipole moment of the MOI, it is of note that solute type and position do affect the calculated HOMO‐LUMO energy gap, which is important for technologies such as photothermal therapy [36] and polymer solar cells [37]. Experimentally, the optical gap of the MOI was found to be 3.0 eV in THF [14], something not broadly agreeable with computations of the MOI infinitely separated from the solvent. Most systems similarly hold a HOMO‐LUMO gap within 4.0 ± 0.25 eV, with the notable exception of the cn2 and cn2cn2′ THF systems, in which the HOMO‐LUMO gap drops significantly, as seen in Tables 7 and 8.

**TABLE 7:** HOMO‐LUMO gaps of water systems in eV.

Method	MOI	nh1	nh1nh1′	cn2	cn2cn2′	SMD
B3LYP/aug‐cc‐pVTZ	4.01	3.91	3.85	4.02	4.02	3.76
B3LYP/def2‐TZVP	4.02	3.92	3.85	4.02	4.05	
B3LYP‐D3BJ/aug‐cc‐pVTZ	4.02	3.91	3.86	4.02	4.03	

*Note:* MOI refers to the MOI infinitely separated from water.

**TABLE 8:** HOMO‐LUMO gaps of THF systems in eV.

Method	MOI	nh1	nh1nh1′	cn2	cn2cn2′	ring	SMD
B3LYP/aug‐cc‐pVTZ	4.01	3.92	3.82	3.01	2.95	4.01	3.82
B3LYP/def2‐TZVP	4.02	3.93	3.83	3.01	2.93	4.01	
B3LYP‐D3BJ/aug‐cc‐pVTZ	4.02	3.91	3.82	2.95	2.87	4.01	

*Note:* MOI refers to the MOI infinitely separated from THF.

### 3.3 Dipole

**FIGURE 5:**
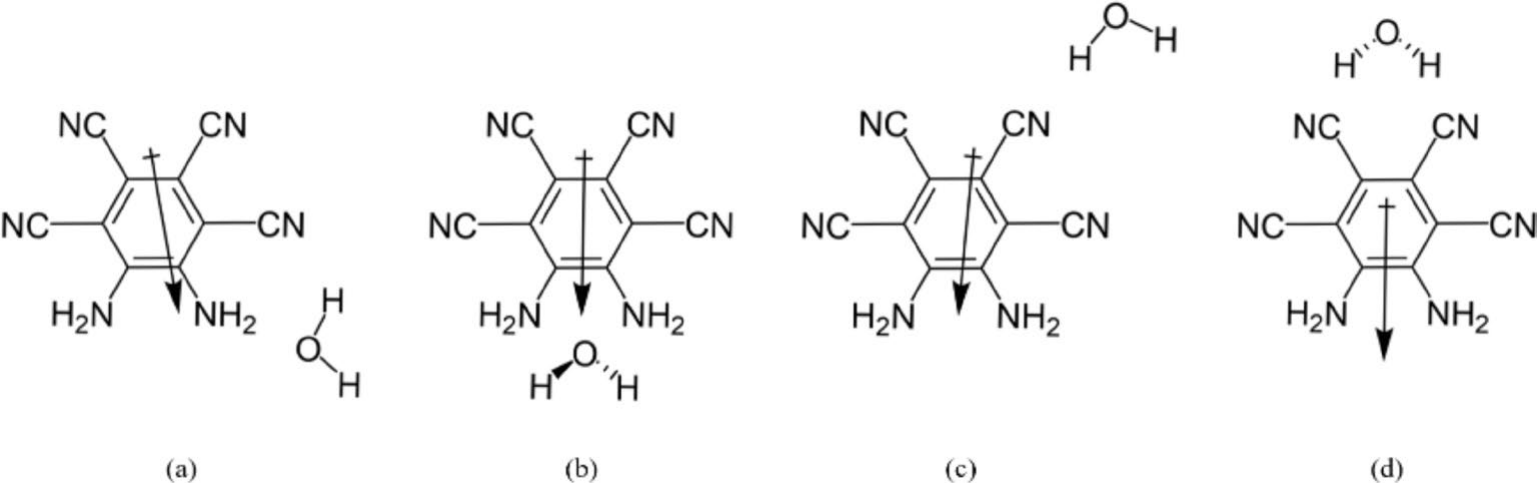
Directions of dipole moments in the one water systems. Not reflective of magnitude. (a) nh1, (b) nh1nh1′, (c) cn2, (d) cn2cn2′.

Dipole moments for the water complexes can be seen in Table 9 and are visualized in Figure 5. Remarkably, placing a single molecule of water in the nh1nh1′ position resulted in a dipole moment within the range of the experimental dipole moment of the MOI in THF. The second‐highest dipole moment achieved with one water molecule can be seen for the cn2 complex, though as seen in Table 3, this system has a low dissociation energy. Using the SMD model for water yields the highest dipole moment for the MOI at 16.6 Debye, as expected from a highly polar solvent.

**TABLE 9:** Dipole moments in debye of the one water systems.

Method	MOI	nh1	nh1nh1′	cn2	cn2cn2′	SMD
B3LYP/aug‐cc‐pVTZ	9.6	10.2	14.2	11.0	10.5	16.6
B3LYP/def2‐TZVP	9.7	10.4	14.6	12.6	10.6	
B3LYP‐D3BJ/aug‐cc‐pVTZ	9.6	10.2	14.2	12.2	8.5	
DLPNO‐CCSD(T)/CBS (Psi4)	9.1	9.7	13.6	10.5	9.9	
DLPNO‐CCSD/aug‐cc‐pVDZ (Orca)	9.1	9.8	13.6	10.5	9.9	
DLPNO‐CCSD/aug‐cc‐pVDZ (Psi4)	9.1	9.3	13.5	10.4	9.8	

*Note:* MOI calculations reflect the dipole moment of the MOI infinitely separated from water. Direction of dipole can be seen in Figure 5.

**FIGURE 6:**
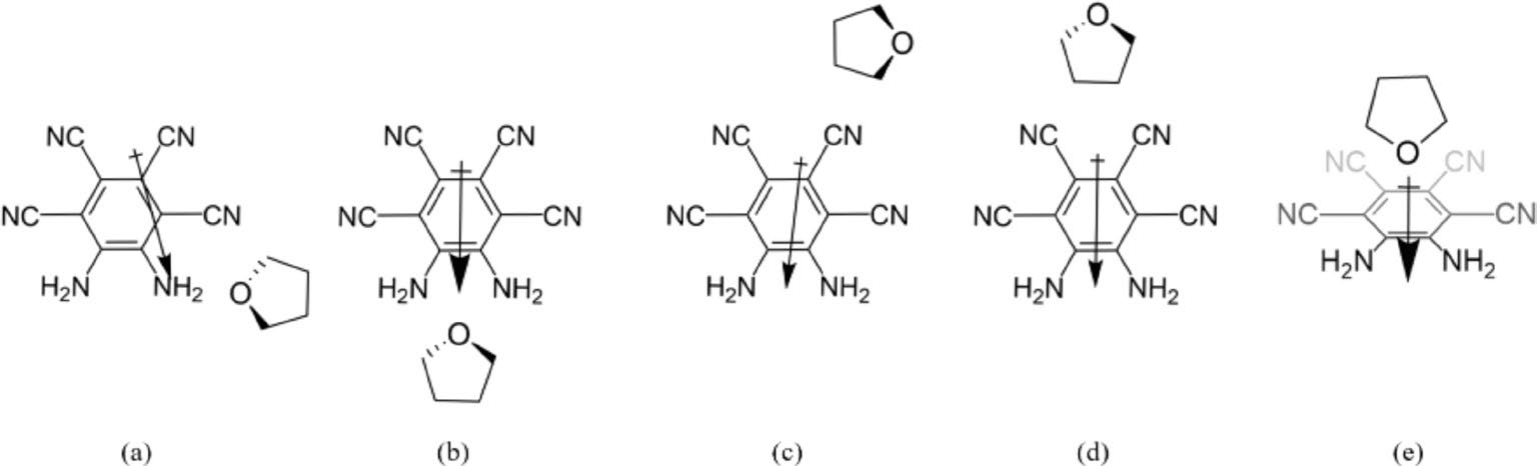
Directions of dipole moments in the THF systems. Not reflective of magnitude. (a) nh1, (b) nh1nh1′, (c) cn2, (d) cn2cn2′, (e) ring.

Dipole moments for the THF systems are visualized in Figure 6, with the dipole moments in Table 10. It is seen that the highest dipole moment is achieved for the nh1nh1′ structure, actually superseding the SMD model for THF. Of all the singly solvated systems, the THF nh1nh1′ system predicts the highest dipole moment, as well as the highest dissociation energy, as seen in Table 4. The nh1 complex also shows a high dipole moment with THF as the solvent, though the cn2cn2′ position holds the second‐largest dipole moment among the singly solvated THF systems. The only complex where the addition of a solvent consistently decreases the dipole moment of the MOI relative to the MOI in gas phase is at the ring position (Figure 6e).

**TABLE 10:** Dipole moments in debye of the one THF complexes.

Method	MOI	nh1	nh1nh1′	cn2	cn2cn2′	ring	SMD
B3LYP/aug‐cc‐pVTZ	9.6	11.2	15.7	10.0	12.1	8.8	15.0
B3LYP/def2‐TZVP	9.7	11.2	15.8	10.1	12.2	9.0	
B3LYP‐D3BJ/aug‐cc‐pVTZ	9.6	11.0	15.8	10.1	12.2	8.8	
DLPNO‐CCSD(T)/CBS	9.1	10.7	15.0	9.5	11.6	8.4	
DLPNO‐CCSD/aug‐cc‐pVDZ (Orca)	9.1	10.6	15.0	9.5	11.6	8.4	
DLPNO‐CCSD/aug‐cc‐pVDZ (Psi4)	9.1	10.6	14.9	9.3	11.5	8.3	

*Note:* MOI calculations reflect the dipole moment of the MOI infinitely separated from THF. Direction of dipole can be seen in Figure 6.

Specifically comparing the effect of solvent on the dipole moment at the nh1 and nh1nh1′ structures, it can be seen that across all levels of theory presented, the THF complexes consistently show a higher dipole moment than water for the analogous positions. This is somewhat counterintuitive, considering that experimentally the dipole moments of THF and water in the gas phase are 1.63 and 1.85 Debye, respectively [38]. The fact that a single THF molecule is able to induce a greater dipole moment than water for these two systems merits further exploration, specifically into the potential hydrogen bonding that could explain how THF, with its lower dipole moment in gas phase, can induce the highest dipole moment of all the singly solvated systems.

Using both THF and water as the solvent, it can be seen that the nh1nh1′ system consistently yields the highest dipole moment, with the THF complex being the highest. It is also of note that while the water SMD model yielded the highest dipole moment overall, the nh1nh1′ water system was the only dipole moment in the observed range of 14.1±0.7 Debye, despite the large margin of experimental error.

While placing a solvent molecule at the cn2 and cn2cn2′ positions resulted in low dissociation energies, these systems could yield surprisingly high dipole moments, as seen in the cn2 water and cn2cn2′ THF systems.

### 3.4 Hydrogen Bonding

Of the systems examined, it is evident that the nh1 and nh1nh1′ positions are those best suited for hydrogen bonding.

Hydrogen bonds are often divided into three categories: strong, moderate, and weak [39]. They can be distinguished via a number of metrics, including A–H‧‧‧‧B distances, wherein A represents the highly electronegative atom partially deshielding hydrogen and B represents the acceptor atom [39]. Strong hydrogen bonding is associated with H‧‧‧‧B distances between 1.2–1.5 Å, and A‧‧‧‧B distances between 2.2 and 2.5 Å. Moderate hydrogen bonds are classified as having H‧‧‧‧B distances between 1.5–2.2 Å, and A‧‧‧‧B distances between 2.5 and 3.2 Å [39].

**FIGURE 7:**
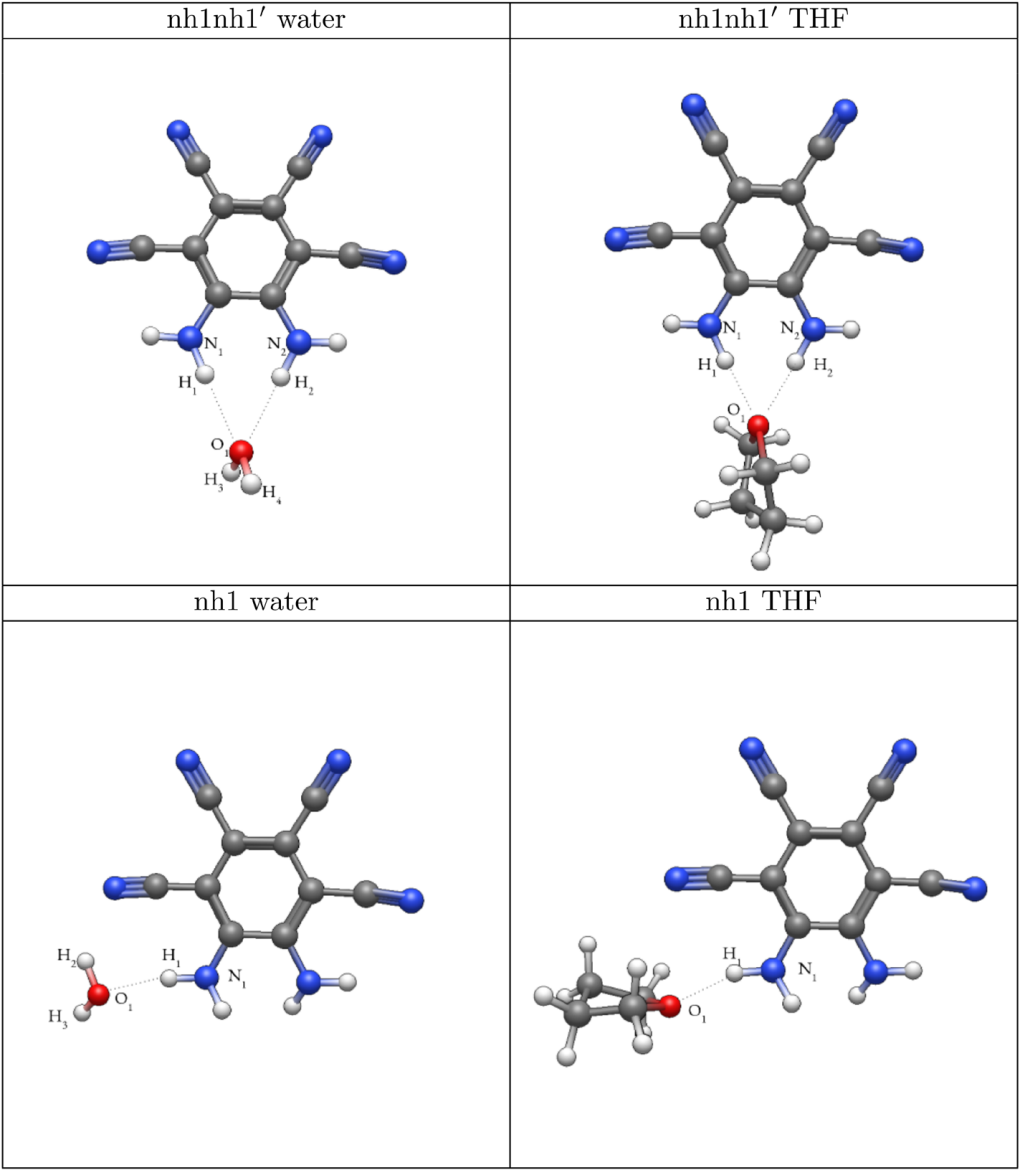
Indexing for atomic distances displayed in Table 11.

Atomic distances for the nh1 and nh1nh1′ systems with both water and THF are depicted in Table 11, and visualized in Figure 7. In the nh1 systems, the N_1_‧‧O_1_ distances were 2.97 and 2.87 Å for water and THF, respectively, well within the A‧‧‧‧B range associated with moderate hydrogen bonding. Furthermore, the H_1_‧‧O_1_ distances were 1.97 Å in the water system and 1.90 Å in the THF system. It is interesting to note that these distances were consistently shorter for the THF system. In the nh1nh1′ systems, the N_1_‧‧O_1_ and N_2_‧‧O_1_ distances were within 0.01 Å of each other. For the water system, these values were 3.05 and 3.06 Å, and for THF they were both 2.98 Å, all of which were longer than the N_1_‧‧O_1_ distance in the nh1 systems. The H_1_‧‧O_1_ and H_3_‧‧O_1_ distances were similarly within 0.01 Å of each other, with the water systems having distances of 2.07 and 2.08 Å, and the THF systems distances of 1.97 and 1.98 Å. While these distances are also longer than the respective distances in the nh1 systems, they are between the solvent and both amine substituents. Thus, they are indicative of not one, but two moderate hydrogen bonds.

**TABLE 11:** Atomic distances in Angstroms. Relevant indexing can be seen in Figure 7.

System	N_1_‧‧O_1_	N_2_‧‧O_1_	O_1_‧‧H_1_	O_1_‧‧H_2_	O_1_‧‧H_3_	O_1_‧‧H_4_
nh1nh1′ water	3.05	3.06	2.07	2.08	0.96	0.96
nh1nh1′ THF	2.98	2.98	1.98	1.97		
nh1 water	2.97		1.97		0.97	0.96
nh1 THF	2.87		1.90			

Reflecting on the dissociation energies presented in Tables 3 and 4, it may be seen for both the water and THF systems that nh1 and nh1nh1′ systems hold the highest two dissociation energies, a logical byproduct of the proposed hydrogen bonding. It is also of note that under the B3LYP/aug‐cc‐pVTZ level of theory, we find the computed dissociation energy of the water dimer, a system well known for its hydrogen bonding, to be 4.82 kcal mol^−1^. With both THF and water as the solvent, the nh1 and nh1nh1′ positions hold a higher dissociation energy than the water dimer at a comparable level of theory. Interaction energies for the nh1 and nh1nh1′ systems, which can also be associated with the strength of a hydrogen bond [40], were similarly found to be over 10 kcal mol^−1^ with the D3BJ dispersion correction (Table 12).

**TABLE 12:** Interaction energies, in kcal mol^−1^, obtained at the B3LYP‐D3BJ/aug‐cc‐pVTZ level of theory.

Solvent	nh1	nh1nh1′	cn2	cn2cn2′	ring
water	−10.84	−11.70	−4.36	−5.78	
THF	−13.05	−15.29	−1.53	−2.42	−8.58

Thus, while none of the O_1_‧‧‧N or O_1_‧‧‧H bond distances were predicted to be within the range of strong hydrogen bonds, all systems are well within the expected range for moderate hydrogen bonds, with one amine substituent in the nh1 systems and with both amine substituents in the nh1nh1′ systems. Across all systems, the nh1nh1′ THF system is predicted to have the highest dissociation energy. In conjunction with its A—H‧‧‧‧B distances being within the range of moderate hydrogen bonding with both amine substituents, this research supports the idea that there is significant hydrogen bonding transpiring, which would help explain the high dipole moments of this system.

### 3.5 Mulisolvated Systems

**FIGURE 8:**
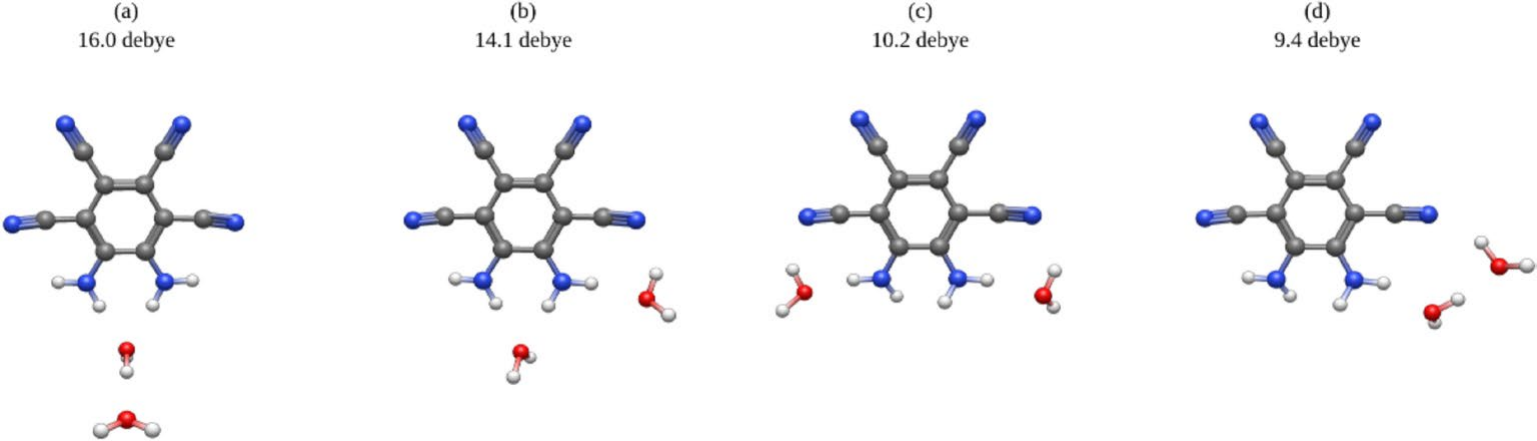
Doubly solvated water systems, dipole moment computed at the B3LYP/aug‐cc‐pVTZ level of theory: (a) 16.0 Debye, (b) 14.1 Debye, (c) 10.2 Debye, (d) 9.4 Debye.

**FIGURE 9:**
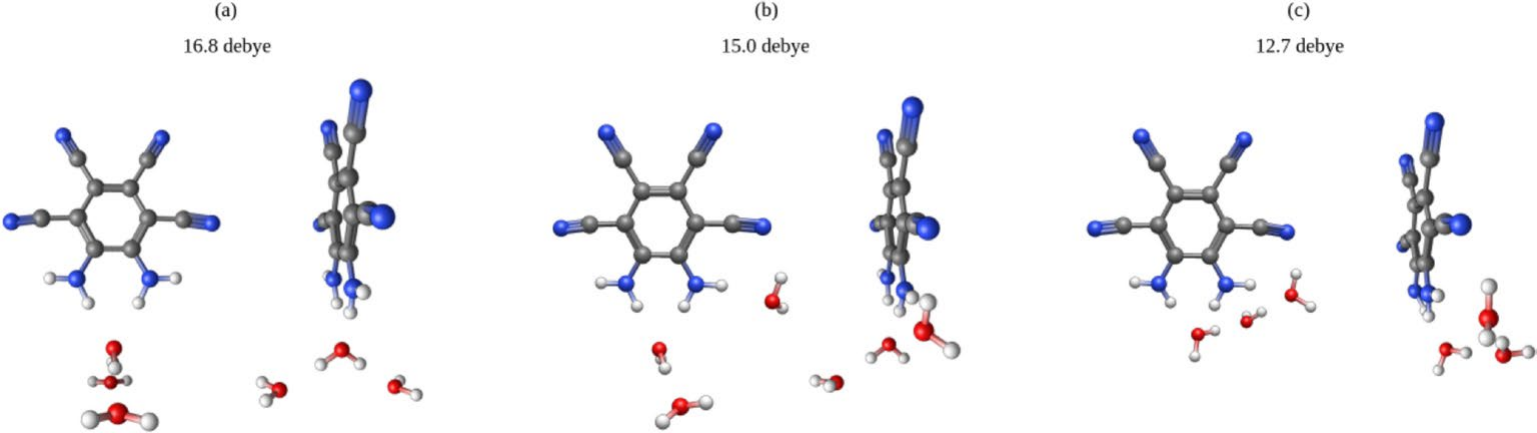
Triply solvated water systems, dipole moment computed at the B3LYP/aug‐cc‐pVTZ level of theory: (a) 16.8 Debye, (b) 15.0 Debye, (c) 12.7 Debye.

**FIGURE 10:**
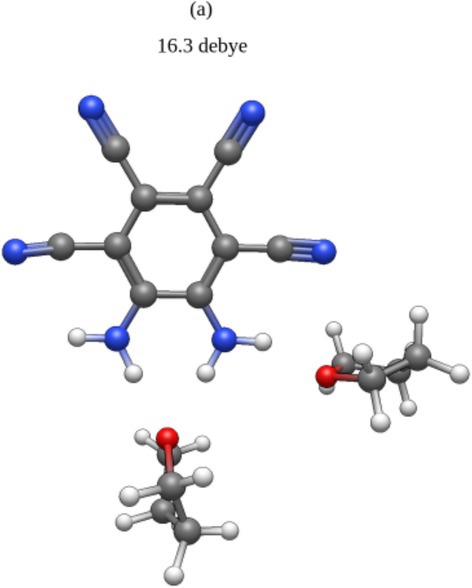
Doubly solvated THF system, dipole moment computed at the B3LYP/aug‐cc‐pVTZ level of theory: (a) 16.3 Debye.

Preliminary explorations of the MOI with multiple solvent molecules are depicted below (Figures 8, 9, 10). Consistent with the high dipole moments afforded by a single solvent molecule at the nh1nh1′ position, it is seen that stacking water molecules at this position will further increase the overall dipole moment. It is notable that those systems which boast the highest dipole moments adopt a conformation suggestive of hydrogen bonding between the water molecules (Figures 8 and 9).

Though THF may induce a higher dipole moment than water with a low number of solvent molecules, water may induce a higher dipole moment with more solvent molecules. These structures suggest that this is a consequence of hydrogen bonding between water molecules, reflecting the SMD results reported in Tables 9 and 10.

These preliminary data do support that the nh1nh1′ position holds the most potential to maximize the MOI's ultrastrong dipole moment and that hydrogen bonding plays an important role in increasing the MOI's dipole moment.

## 4 Conclusions

While the dipole moment of the MOI is quite high in gas phase theoretical computations, it is significantly lower than the solvent phase experimental result from Müllen et al. [14] This shows that the solvent plays a significant role in increasing this dipole moment, and thus must be considered for a fuller understanding of the MOI in solution. In this paper, the importance of solvent molecule position in relation to the MOI is explored, through the strategic placement of water and THF about the MOI. It is seen that placing a solvent molecule at the nh1nh1′ position results in the highest possible dipole moment with both water and THF as the solvent. These systems also boast the highest dissociation energy, which can be attributed to the hydrogen bonding occurring at these positions. Furthermore, it is of note that with the nh1 and nh1nh1′ complexes, THF participates in stronger hydrogen bonding than water, thus inducing a higher dipole moment than water for each system, respectively. However, with the addition of two and three water molecules, water's ability to hydrogen bond with itself allows for notably higher dipole moments to be induced, up to 16.8 Debye as seen in Figure 9. It is also of note that while the cn2cn2′ THF system did not boast the highest dipole moment, it is still consistently predicted to yield an increase in dipole of over 2 Debye relative to the MOI in gas phase. This complex also displays the lowest HOMO‐LUMO energy gap. Finally, it is noteworthy that despite the large margin of error in the experimental dipole moment, the only system to show a dipole moment within this experimental range was the nh1nh1′ water system and the doubly hydrated system b.

Reflecting on the peculiarly high dipole moments achieved with a single THF molecule at the nh1 and nh1nh1′ positions relative to water, a future rigorous exploration of multisolvated complexes may be merited. For synthesizing organic frameworks or other macromolecules using the MOI or MOI‐like subunits, understanding the impact of solvent position might prove beneficial. Finally, it is noteworthy that no THF system, including the SMD model, was able to predict a dipole moment for the MOI within the proposed experimental error. Future explicit solvation exploration using a larger number of THF molecules to better understand the solvent phase dipole of the MOI in THF might be fruitful.

## Conflicts of Interest

The authors declare no conflicts of interest.

## Supporting information


**Data S1.** Supporting Information.

## Data Availability

The data that supports the findings of this study are available in the Supporting Information of this article.
