# Supplementary material for: Giant Dipole Moments: Remarkable Effects Mono‐, Di‐, and Tri‐ Hydrated 5,6‐Diaminobenzene‐1,2,3,4‐Tetracarbonnitrile
Source: J Comput Chem. 2025 Apr 18;46(11):e70105. doi: 10.1002/jcc.70105 (PMC12008715; doi:10.1002/jcc.70105)
Supplement: Supplementary file 1 — Data S1. Supporting Information. [file JCC-46-0-s001.pdf]

8 Supporting Information

8.1 Bond Orders

Figure 11 Atom Indexing of the MOI.

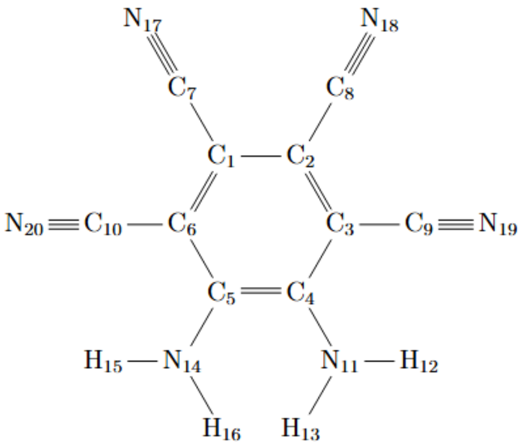

Table 13 Bond orders of the MOI, with the addition of either THF or water at various positions. MOI Indexing can be seen in Figure 11. MOI indicates the bond order of the MOI infinitely separated from either solvent.

	MOI	nh1		nh1nh1′		cn2		cn2cn2′		ring	
Bond		Water	THF	Water	THF	Water	THF	Water	THF	Water	THF
C1-C2	1.46	1.46	1.47	1.45	1.47	1.45	1.50	1.45	1.50		1.50
C1-C7	1.07	1.08	1.07	1.07	1.07	1.05	1.10	1.07	1.10		1.10
C1-C5	1.33	1.32	1.33	1.32	1.32	1.33	1.33	1.33	1.33		1.33
C2-C3	1.33	1.31	1.32	1.31	1.32	1.33	1.33	1.33	1.33		1.33
C2-C8	1.07	1.07	1.07	1.07	1.07	1.07	1.10	1.07	1.10		1.10
C3-C4	1.40	1.39	1.39	1.39	1.40	1.39	1.38	1.40	1.38		1.38
C3-C9	1.11	1.11	1.11	1.13	1.13	1.11	1.10	1.11	1.10		1.10
C4-C5	1.26	1.23	1.24	1.21	1.20	1.25	1.28	1.25	1.28		1.28
C4-N11	1.25	1.27	1.27	1.26	1.24	1.26	1.24	1.25	1.24		1.24
C5-C6	1.40	1.40	1.41	1.39	1.40	1.40	1.37	1.40	1.37		1.37
C5-N14	1.25	1.25	1.24	1.25	1.24	1.25	1.24	1.25	1.24		1.24
C6-C10	1.11	1.10	1.10	1.14	1.13	1.11	1.10	1.11	1.10		1.10
C7-N17	2.86	2.85	2.87	2.86	2.87	2.90	2.84	2.86	2.84		2.83
C8-N18	2.86	2.86	2.88	2.86	2.87	2.86	2.84	2.86	2.84		2.83
C9-N19	2.82	2.82	2.83	2.80	2.82	2.82	2.84	2.82	2.84		2.84
C10-N20	2.82	2.83	2.85	2.79	2.82	2.82	2.84	2.82	2.84		2.84
N11-H12	0.99	0.96	0.98	1.00	0.99	0.99	0.99	0.99	0.99		0.99
N11-H13	0.99	0.99	0.99	0.99	0.98	0.99	0.99	0.99	0.99		0.99
N14-H15	0.99	0.98	0.99	0.99	0.99	0.99	0.99	0.99	0.99		0.99
N14-H16	0.99	0.99	0.99	0.98	0.98	0.99	0.99	0.99	0.99		0.99

8.2 Atomic Charges

Table 14 Atomic charge of the MOI, with the addition of either THF or water at various positions. Relevant indexing of the MOI can be seen in Figure 11. MOI indicates the bond order of the MOI infinitely separated from either solvent.

	MOI	nh1		nh1nh1′		cn2		cn2cn2′		ring	
Atom		Water	THF	Water	THF	Water	THF	Water	THF	Water	THF
C1	–0.07	–0.09	–0.09	–0.08	–0.08	–0.8	–0.07	–0.07	–0.07		–0.07
C2	–0.07	–0.06	–0.06	–0.08	–0.08	–0.06	–0.07	–0.07	–0.07		–0.07
C3	–0.17	–0.20	–0.20	–0.19	–0.2	–0.17	–0.17	–0.17	–0.17		–0.16
C4	0.26	0.29	0.29	0.28	0.28	0.27	0.26	0.26	0.26		0.27
C5	0.26	0.25	0.25	0.28	0.28	0.26	0.26	0.26	0.26		0.27
C6	–0.17	–0.17	–0.17	–0.19	–0.2	–0.16	–0.17	–0.16	–0.17		–0.17
C7	0.27	0.28	0.28	0.28	0.28	0.32	0.27	0.30	0.28		0.28
C8	0.27	0.27	0.28	0.28	0.28	0.27	0.28	0.30	0.28		0.28
C9	0.29	0.34	0.31	0.30	0.30	0.28	0.29	0.28	0.29		0.29
C10	0.29	0.29	0.29	0.30	0.30	0.28	0.29	0.28	0.29		0.29
N11	–0.82	–0.84	–0.84	–0.83	–0.84	–0.82	–0.82	–0.82	–0.82		–0.82
H12	0.41	0.45	0.46	0.41	0.41	0.41	0.41	0.41	0.41		0.41
H13	0.39	0.39	0.39	0.43	0.44	0.40	0.39	0.40	0.39		0.39
N14	–0.82	–0.82	–0.82	–0.83	–0.84	–0.82	–0.82	–0.82	–0.82		–0.82
H15	0.41	0.41	0.41	0.41	0.41	0.41	0.41	0.41	0.41		0.41
H16	0.39	0.39	0.39	0.43	0.44	0.39	0.39	0.40	0.39		0.39
N17	–0.27	–0.27	–0.28	–0.28	–0.28	–0.32	–0.27	–0.30	–0.27		–0.28
N18	–0.27	–0.27	–0.27	–0.28	–0.28	–0.26	–0.28	–0.30	–0.27		–0.28
N19	–0.30	–0.36	–0.33	–0.32	–0.32	–0.29	–0.29	–0.29	–0.3		–0.31
N20	–0.30	–0.30	–0.30	–0.32	–0.32	–0.29	–0.30	–0.29	–0.3		–0.31

8.3 Coordinates

Final Cartesian coordinates in angstroms presented are from the B3LYP/aug-cc-pVTZ optimizations.

8.4 1 Water Systems

nh1:

C	-1.053660	0.520465	-0.266177
C	-0.054496	1.423143	-0.622584
C	1.301037	1.044552	-0.474525
C	1.660035	-0.206500	0.034907
C	-0.700594	-0.738804	0.274931
C	0.641019	-1.113326	0.456249
C	-2.429034	0.858522	-0.425994
C	-0.380473	2.705691	-1.149249
C	2.354116	1.918097	-0.872094
C	-1.715147	-1.648367	0.684463
N	2.973351	-0.580303	0.191310
H	3.668531	0.047117	-0.185322
H	3.185891	-1.552995	0.030189
N	1.016802	-2.313779	0.976539
H	0.331316	-2.967828	1.349483
H	1.901588	-2.342736	1.456879
N	-3.544774	1.116203	-0.551155
N	-0.629804	3.746480	-1.575677
N	3.272203	2.549385	-1.168532
N	-2.482172	-2.430494	1.042259
O	-0.679046	-4.374417	2.281831
H	-0.673437	-5.331175	2.185509
H	-1.581838	-4.089114	2.081057

nh1nh1′ :

C	-1.340482	0.566834	-0.390448
C	-0.221316	1.373152	-0.580505
C	1.055940	0.876725	-0.230603
C	1.235104	-0.407255	0.308385
C	-1.175781	-0.727485	0.155107
C	0.085415	-1.231863	0.511026
C	-2.643006	1.030087	-0.733781
C	-0.347127	2.683577	-1.124476
C	2.225184	1.666110	-0.421033
C	-2.296530	-1.577166	0.375030
N	2.470724	-0.851202	0.662097
H	3.268936	-0.280789	0.436077
H	2.658944	-1.818755	0.868028
N	0.220275	-2.488890	1.010420
H	-0.611543	-3.019497	1.210334
H	1.071176	-2.812846	1.440549
N	-3.702351	1.391511	-1.007009
N	-0.433182	3.745495	-1.563145
N	3.232444	2.216478	-0.53483
N	-3.130654	-2.342618	0.595858
O	2.928452	-3.641883	1.823454
H	3.170256	-4.469140	1.393712
H	3.360260	-3.654709	2.684206

cn2:

C	-0.220711	0.230929	0.527174
C	1.120099	-0.115140	0.696871
C	1.696321	-1.089037	-0.148783
C	0.947427	-1.724809	-1.147884
C	-0.990446	-0.425036	-0.460145
C	-0.432617	-1.410510	-1.283897
C	-0.821870	1.225193	1.351226
C	1.913106	0.514917	1.699076
C	3.072478	-1.439573	-0.037092
C	-2.372839	-0.128848	-0.634312
N	1.489917	-2.677440	-1.971431
H	2.486693	-2.822456	-1.909346
H	1.131165	-2.722412	-2.912606
N	-1.161796	-2.050937	-2.254229
H	-2.153603	-1.865025	-2.275521
H	-0.929149	-3.015677	-2.433098
N	-1.315556	2.024085	2.015873
N	2.558895	1.017194	2.509908
N	4.171962	-1.786260	-0.032001
N	-3.493343	0.036744	-0.849006
O	-2.726524	4.022180	3.920867
H	-2.038818	4.328378	4.518370
H	-2.277045	3.423648	3.309070

cn2cn2′ :

C	-0.894069	0.128657	0.233332
C	0.259019	0.896607	0.068189
C	1.525686	0.287625	0.208215
C	1.650365	-1.072314	0.519762
C	-0.777081	-1.236275	0.576929
C	0.475350	-1.841537	0.742379
C	-2.180643	0.717302	0.067667
C	0.162184	2.278785	-0.262464
C	2.724937	1.029463	0.006727
C	-1.931451	-2.040769	0.798696
N	2.872048	-1.679580	0.666450
H	3.684638	-1.132448	0.423617
H	2.934991	-2.643983	0.378498
N	0.608696	-3.168047	1.065549
H	-0.236504	-3.669735	1.294434
H	1.396349	-3.417163	1.643682
N	-3.216077	1.201101	-0.075792
N	0.073458	3.394043	-0.536421
N	3.746651	1.543941	-0.136377
N	-2.802314	-2.767635	1.004503
O	-2.218392	2.723348	-2.901465
H	-1.619492	3.312536	-2.429855
H	-2.875353	2.466714	-2.245113

8.5 1 THF Systems

nh1:

C	-0.996697	0.030475	-0.513164
C	-0.112459	1.069793	-0.794911
C	1.230119	0.967982	-0.358777
C	1.682006	-0.138692	0.364651
C	-0.555915	-1.084459	0.238470
C	0.763780	-1.175642	0.711913
C	-2.348113	0.088529	-0.963380
C	-0.535954	2.213909	-1.529486
C	2.181178	1.982679	-0.668846
C	-1.465173	-2.133816	0.552447
N	2.982237	-0.241815	0.803103
H	3.621588	0.469230	0.479794
H	3.384667	-1.166738	0.796399
N	1.214266	-2.224247	1.448952
H	0.588165	-2.948666	1.805670
H	2.006166	-2.056150	2.047288
N	-3.441894	0.124244	-1.322949
N	-0.864111	3.146057	-2.121909
N	3.022524	2.744250	-0.872752
N	-2.164447	-3.007148	0.829645
O	0.065377	-4.476839	2.808696
C	-0.162298	-5.701576	2.074751
C	-0.882026	-4.369802	3.895084
H	-0.338883	-4.059613	4.788032
H	-1.620677	-3.605555	3.641556
C	-1.529663	-5.745105	4.023249
H	0.665287	-6.386465	2.280814
C	-1.492993	-6.253320	2.576653
H	-0.169672	-5.467912	1.010408
H	-2.316086	-5.827157	2.003382
H	-1.544212	-7.338607	2.502633
H	-0.934986	-6.389884	4.672969
H	-2.537743	-5.688646	4.430813

nh1nh1′ :

C	-1.594347	0.622755	-0.364864
C	-0.549997	1.510308	-0.607773
C	0.778180	1.108764	-0.330094
C	1.081780	-0.161207	0.186735
C	-1.304888	-0.660315	0.156819
C	0.007389	-1.072931	0.439448
C	-2.944178	0.989911	-0.632940
C	-0.801049	2.810742	-1.131699
C	1.873658	1.985443	-0.568298
C	-2.344780	-1.594611	0.423572
N	2.361605	-0.520190	0.456466
H	3.104594	0.124336	0.245006
H	2.623305	-1.449432	0.753581
N	0.261645	-2.311297	0.930615
H	-0.509326	-2.924594	1.134494
H	1.183835	-2.616972	1.206365
N	-4.040914	1.272871	-0.844664
N	-0.988629	3.865742	-1.555410
N	2.830297	2.610986	-0.724333
N	-3.105393	-2.424456	0.675796
O	3.041180	-3.209873	1.539870
C	3.630787	-4.265089	0.736559
C	3.556798	-3.269452	2.895114
H	4.328810	-3.811198	0.029051
C	4.343275	-5.185981	1.721065
H	2.833321	-4.753509	0.177974
H	3.815112	-2.257478	3.204335
H	2.767881	-3.647482	3.549977
C	4.748014	-4.218766	2.840714
H	5.191965	-5.694593	1.267367
H	3.657138	-5.944429	2.100939
H	5.655696	-3.677511	2.569421
H	4.922318	-4.715654	3.793379

cn2:

C	-0.393602	-0.366032	-0.002836
C	0.623125	0.298080	-0.689383
C	1.970948	-0.034209	-0.424579
C	2.311039	-1.009085	0.521945
C	-0.057335	-1.331662	0.972824
C	1.276897	-1.646512	1.259620
C	-1.762590	-0.067591	-0.262809
C	0.315628	1.291248	-1.663820
C	3.036125	0.590200	-1.135173
C	-1.065873	-2.000917	1.724045
N	3.615064	-1.336909	0.797958
H	4.322091	-0.930748	0.203173
H	3.797475	-2.298158	1.042231
N	1.621006	-2.588661	2.197000
H	0.872556	-2.963355	2.761026
H	2.470037	-2.421346	2.714850
N	-2.872290	0.163383	-0.467239
N	0.078298	2.094230	-2.454852
N	3.963843	1.034328	-1.656015
N	-1.805247	-2.591456	2.382647
O	-8.120294	1.062734	-0.247872
C	-8.762834	1.103805	-1.528168
C	-6.695028	1.162662	-0.407453
H	-6.220036	0.399939	0.209378
H	-6.365987	2.145804	-0.054103
C	-6.416919	0.987878	-1.899486
C	-7.697164	1.548671	-2.529730
H	-9.143275	0.105007	-1.772032
H	-9.611660	1.787344	-1.467728
H	-5.512028	1.503111	-2.216501
H	-6.303629	-0.070124	-2.143001
H	-7.885189	1.172763	-3.535115
H	-7.651706	2.638592	-2.578572

cn2cn2$'$:

C	-0.452805	-0.044008	0.164735
C	0.696767	0.646618	-0.219354
C	1.949566	-0.004885	-0.163538
C	2.065706	-1.327926	0.281206
C	-0.339947	-1.369893	0.640610
C	0.901552	-2.012764	0.724281
C	-1.732083	0.580059	0.097911
C	0.616004	1.992024	-0.681793
C	3.140164	0.652087	-0.587985
C	-1.484954	-2.092875	1.082830
N	3.275359	-1.972695	0.350775
H	4.070339	-1.493344	-0.045184
H	3.270258	-2.964401	0.168131
N	1.028316	-3.303179	1.174705
H	0.200382	-3.735048	1.557582
H	1.870138	-3.523285	1.684413
N	-2.769968	1.076570	0.043721
N	0.558960	3.080221	-1.054928
N	4.154179	1.094627	-0.912139
N	-2.347242	-2.755255	1.466047
O	-4.429728	6.816547	-1.932815
C	-4.897945	5.461164	-1.870183
C	-3.038679	6.871575	-1.581603
H	-2.893682	7.687295	-0.871160
H	-2.452357	7.091092	-2.480773
C	-2.683331	5.500119	-1.008700
C	-3.653991	4.581255	-1.760277
H	-5.544791	5.345269	-0.993188
H	-5.492849	5.261050	-2.762884
H	-1.637714	5.236732	-1.159460
H	-2.889788	5.470004	0.063125
H	-3.844296	3.642056	-1.243429
H	-3.262850	4.350039	-2.753212

ring:

C	1.470880	0.671599	-0.231716
C	1.474624	-0.720901	-0.166410
C	0.983045	-1.362923	0.991799
C	0.471477	-0.631466	2.069778
C	0.934453	1.414931	0.843188
C	0.412096	0.784600	1.977231
C	1.969930	1.348039	-1.381951
C	1.969418	-1.499773	-1.251989
C	1.006096	-2.781731	1.113698
C	0.860131	2.836226	0.788047
N	-0.024251	-1.238198	3.196816
H	0.111707	-2.234915	3.275187
H	0.099573	-0.743290	4.066541
N	-0.117850	1.489263	3.031591
H	-0.228512	2.483327	2.895528
H	-0.907433	1.063880	3.494037
N	2.366225	1.908119	-2.307436
N	2.358451	-2.141686	-2.125969
N	0.984267	-3.918125	1.308029
N	0.748860	3.983048	0.832342
O	-2.046359	0.047382	-0.621633
C	-2.468268	-1.180568	-1.221257
C	-2.281417	1.061643	-1.601671
H	-1.466995	1.064610	-2.336557
H	-2.292197	2.022973	-1.091159
C	-3.616352	0.692934	-2.263238
C	-3.718644	-0.839657	-2.051396
H	-1.669356	-1.572352	-1.862192
H	-2.653253	-1.894278	-0.420379
H	-4.441625	1.205334	-1.770803
H	-3.634138	0.971489	-3.315776
H	-3.729860	-1.387586	-2.992352
H	-4.627842	-1.096066	-1.509974

8.6 2 Water Systems

a.

C	0.748763	-1.806461	-0.282273
C	1.763978	-0.888419	-0.030197
C	1.441538	0.484378	0.086934
C	0.124728	0.957692	-0.039297
C	-0.582554	-1.345874	-0.415594
C	-0.920085	0.012915	-0.298679
C	1.032994	-3.196379	-0.407351
C	3.116304	-1.312647	0.109738
C	2.452428	1.452657	0.343312
C	-1.647122	-2.254069	-0.674583
N	-0.157661	2.276569	0.099623
H	0.601597	2.923362	0.232531
H	-1.071268	2.672442	-0.068019
N	-2.207881	0.422811	-0.408714
H	-2.914206	-0.255255	-0.640703
H	-2.485228	1.393600	-0.419757
N	1.247236	-4.323881	-0.510546
N	4.214769	-1.640912	0.225614
N	3.189763	2.315159	0.551422
N	-2.579863	-2.901013	-0.880343
O	-4.324745	4.270416	1.898853
H	-5.016911	3.751437	2.320976
H	-3.878980	4.745745	2.607368
O	-2.918763	3.309433	-0.299497
H	-3.191811	3.824310	-1.064468
H	-3.421884	3.657197	0.463223

b.

C	0.516577	-1.513526	-0.007482
C	1.692497	-0.772663	0.027953
C	1.614431	0.641138	0.045844
C	0.388880	1.321886	0.038532
C	-0.730636	-0.842092	-0.014258
C	-0.829076	0.561895	0.018376
C	0.551364	-2.937486	-0.034568
C	2.963854	-1.413754	0.039460
C	2.795301	1.436052	0.065281
C	-1.938624	-1.590231	-0.049978
N	0.346828	2.680429	0.086028
H	1.215045	3.186421	0.022020
H	-0.485061	3.204084	-0.133737
N	-2.022139	1.194568	0.026077
H	-2.906537	0.696176	0.022360
H	-2.095649	2.196430	0.081151
N	0.562244	-4.089503	-0.055576
N	4.000483	-1.916921	0.049578
N	3.685479	2.169531	0.077084
N	-2.960369	-2.124020	-0.075970
O	-4.835250	0.113474	-0.041308
H	-4.621076	-0.830365	-0.090307
H	-5.516766	0.190531	0.632711
O	-2.235830	4.260130	-0.208448
H	-2.460274	4.895838	0.479019
H	-2.709988	4.543381	-0.997321

c.

C	0.249885	-1.535903	-0.066255
C	1.377715	-0.726161	0.015882
C	1.222611	0.681852	0.056829
C	-0.042019	1.286338	0.030527
C	-1.036251	-0.940781	-0.063646
C	-1.206553	0.448994	0.006282
C	0.372982	-2.953589	-0.133753
C	2.683751	-1.294752	0.040026
C	2.369532	1.523512	0.086282
C	-2.199308	-1.759997	-0.092991
N	-0.220009	2.636332	0.065321
H	0.573122	3.273565	0.032019
H	-1.041866	2.998405	-0.390544
N	-2.430314	1.047074	0.013913
H	-3.285648	0.496382	0.050918
H	-2.502413	1.928131	0.496539
N	0.455467	-4.101363	-0.190542
N	3.746796	-1.738456	0.061937
N	3.250504	2.266418	0.102965
N	-3.183942	-2.358820	-0.107157
O	-5.130181	-0.193576	0.233959
H	-5.897092	-0.036960	-0.324670
H	-4.936031	-1.139154	0.162171
O	1.809572	4.808873	-0.127307
H	2.645484	4.322354	-0.088099
H	1.915715	5.566007	0.455999

d.

C	0.595402	-1.202644	-0.083257
C	1.878077	-0.668653	0.001777
C	2.037694	0.738279	0.024321
C	0.940077	1.600629	-0.020214
C	-0.526679	-0.338217	-0.103927
C	-0.383551	1.059047	-0.050298
C	0.398491	-2.613567	-0.130473
C	3.022714	-1.514695	0.046366
C	3.334490	1.327655	0.060918
C	-1.836342	-0.890521	-0.135687
N	1.085427	2.966804	0.011427
H	2.029096	3.320893	-0.042130
H	0.427867	3.502182	-0.534626
N	-1.432516	1.917771	-0.059805
H	-2.407988	1.614202	-0.048582
H	-1.293480	2.819558	0.365393
N	0.220808	-3.751075	-0.169536
N	3.956642	-2.188324	0.084063
N	4.337208	1.896349	0.082452
N	-2.910555	-1.304396	-0.149661
O	-5.645779	-0.508346	0.282618
H	-4.860747	-1.058830	0.120989
H	-6.082306	-0.877841	1.055364
O	-4.295769	1.891897	0.046095
H	-4.845302	1.098939	0.220585
H	-4.741060	2.354149	-0.669574

8.7 3 Water Systems

a.

C	0.709661	-1.780189	-0.253912
C	1.728863	-0.870036	0.008078
C	1.420623	0.509336	0.091454
C	0.114084	0.996780	-0.080418
C	-0.612769	-1.306440	-0.431166
C	-0.936833	0.058351	-0.350512
C	0.979890	-3.175452	-0.345496
C	3.071557	-1.307645	0.192078
C	2.435864	1.470179	0.357237
C	-1.681355	-2.206356	-0.701055
N	-0.157304	2.319696	0.011379
H	0.598081	2.960145	0.187915
H	-1.079034	2.714571	-0.132293
N	-2.210181	0.486595	-0.515741
H	-2.928143	-0.188147	-0.718702
H	-2.480936	1.462325	-0.493443
N	1.183326	-4.307155	-0.422416
N	4.162420	-1.647070	0.343286
N	3.178183	2.328502	0.565750
N	-2.616020	-2.845245	-0.923428
O	-4.206631	4.146457	2.044634
H	-4.889747	3.596827	2.441590
H	-3.709795	4.520875	2.779013
O	-2.881424	3.322477	-0.315058
H	-3.161445	3.875155	-1.065470
H	-3.357842	3.637373	0.473262
O	-3.631889	4.868990	-2.563575
H	-3.030001	5.502119	-2.967346
H	-4.143481	4.492434	-3.286509

b.

C	0.718044	-1.811613	-0.143138
C	1.754821	-0.893138	-0.030201
C	1.456057	0.491258	-0.053963
C	0.144944	0.972664	-0.193567
C	-0.612879	-1.344204	-0.282660
C	-0.930887	0.026462	-0.319483
C	0.974290	-3.212313	-0.117091
C	3.104282	-1.325252	0.111517
C	2.489567	1.461789	0.068655
C	-1.686592	-2.269446	-0.382297
N	-0.108287	2.302388	-0.218249
H	0.655848	2.947124	-0.105545
H	-1.037128	2.695639	-0.259651
N	-2.200161	0.460134	-0.471775
H	-2.983648	-0.179621	-0.543711
H	-2.448669	1.437957	-0.441012
N	1.160863	-4.349380	-0.097540
N	4.200218	-1.662140	0.227438
N	3.248413	2.325519	0.164009
N	-2.617211	-2.945915	-0.462707
O	-4.554567	3.550291	1.911928
H	-5.015240	2.738903	2.150511
H	-4.305818	3.969563	2.741862
O	-2.858270	3.384839	-0.282117
H	-3.228750	3.909330	-0.997656
H	-3.449252	3.504476	0.487634
O	-4.839023	-1.053495	-0.531017
H	-4.457875	-1.943757	-0.484075
H	-5.466702	-1.080790	-1.259179

c.

C	1.436776	-1.227981	0.057568
C	2.238169	-0.094594	-0.021664
C	1.639536	1.186886	0.079057
C	0.262966	1.354001	0.286524
C	0.045537	-1.076540	0.274464
C	-0.554768	0.184967	0.440733
C	1.988921	-2.533519	-0.079159
C	3.643407	-0.207161	-0.223978
C	2.424027	2.368108	-0.046923
C	-0.824314	-2.198574	0.304906
N	-0.299425	2.587586	0.383565
H	0.283735	3.386498	0.192234
H	-1.289066	2.723762	0.225527
N	-1.863499	0.312942	0.743865
H	-2.463084	-0.496772	0.775333
H	-2.339364	1.204120	0.717579
N	2.409164	-3.601106	-0.187230
N	4.781418	-0.285982	-0.386666
N	2.968872	3.380360	-0.140724
N	-1.642211	-3.012631	0.312336
O	-4.759950	0.710881	-1.269162
H	-4.921681	0.607823	-2.210901
H	-4.600356	-0.183537	-0.914723
O	-3.321034	2.705066	-0.035561
H	-3.878477	2.085936	-0.556240
H	-3.922682	3.320718	0.392409
O	-4.261869	-1.739517	0.066138
H	-4.973927	-2.121562	0.588264
H	-3.577126	-2.426564	0.001578

8.8 2 THF System

C	-1.325483	0.156401	-0.309811
C	-0.386484	1.065715	-0.784022
C	0.990046	0.804931	-0.577506
C	1.440891	-0.342414	0.092694
C	-0.888877	-1.008649	0.369124
C	0.473588	-1.284440	0.585868
C	-2.718943	0.384059	-0.499265
C	-0.789476	2.246253	-1.470499
C	1.983241	1.708482	-1.048980
C	-1.845681	-1.942222	0.854978
N	2.765714	-0.559862	0.281453
H	3.425087	0.102738	-0.090381
H	3.141906	-1.389387	0.718198
N	0.881946	-2.395893	1.233196
H	0.236508	-3.086255	1.609148
H	1.858064	-2.591820	1.398293
N	-3.848845	0.556489	-0.646168
N	-1.100333	3.205842	-2.027995
N	2.865811	2.369470	-1.388601
N	-2.578915	-2.730921	1.267669
O	-0.257128	-4.739639	2.557389
C	-0.908605	-5.774656	1.786982
C	-0.912444	-4.593942	3.836011
H	-0.141712	-4.506530	4.602450
H	-1.509494	-3.678754	3.824302
C	-1.795855	-5.826949	3.997366
H	-0.227606	-6.627803	1.711627
C	-2.183828	-6.132775	2.545421
H	-1.103269	-5.389120	0.786490
H	-3.001337	-5.486442	2.227555
H	-2.479356	-7.169428	2.390063
H	-1.226466	-6.653431	4.427276
H	-2.655682	-5.636693	4.637853
O	3.812810	-2.973125	1.643142
C	4.354682	-4.12722	0.952861
C	4.373144	-2.881948	2.977089
H	5.038821	-3.775853	0.176529
C	5.079138	-4.94371	2.017618
H	3.529380	-4.658377	0.480283
H	4.664571	-1.847134	3.152783
H	3.599184	-3.157722	3.697551
C	5.541227	-3.861355	3.001338
H	5.901041	-5.524325	1.602347
H	4.388108	-5.633044	2.504988
H	6.451512	-3.379918	2.640613
H	5.735005	-4.245907	4.001190
